# Efficacy of Omalizumab against Japanese Cedar pollinosis in clinical practice

**DOI:** 10.1186/s13223-025-00995-y

**Published:** 2025-11-28

**Authors:** Momoko Takeda, Hiroshi Utsunomiya, Toshio Miki

**Affiliations:** 1 Takeda ENT Clinic, 1-19-5 Nishimizuho-dai, Fujimi City, Saitama 354-0018 Japan; 2 Kanazawa Professional University of Food Management, Yokoe, Hakusan City, Ishikawa 5250 Japan; 3 Department of Physiology, Nihon University School of Medicine, 30-1 Oyaguchi-Kamimachi, Itabashi-ku, Tokyo, 173-8610 Japan

**Keywords:** Omalizumab, Japanese cedar pollinosis, Allergic rhinitis, Nasal eosinophil test, Quality of life, Work productivity

## Abstract

**Background:**

Japanese cedar pollinosis (JCP), which affects over 40% of the population and represents a major public health issue in Japan, has various treatment options, but limited clinical reports and high costs necessitate careful patient selection. This study aimed to evaluate the efficacy of omalizumab in treating JCP and identify key considerations for its appropriate clinical application.

**Methods:**

We retrospectively analyzed 42 patients with JCP treated with omalizumab from 2021 to 2024. Treatment response was assessed using a 3-category patient-reported scale across all years. In a 2023–2024 subset (*n* = 23), quantitative total symptom score (TSS) data were available, allowing effect-size estimation and subgroup analyses.

**Results:**

The study included 42 patients (30 men, 71.4%) aged 12–80 years (mean, 41.5 ± 17.2 years). Symptom improvement was observed in 38 patients (90%), including marked improvement in 23 (55%). Responders were younger (mean age 39.9 vs. 55.5 years) and had higher total IgE levels (251 vs. 211 IU/mL) than nonresponders. No significant correlation was observed between nasal eosinophil counts and treatment response. In the 2023–2024 subset (*n* = 23), TSS decreased significantly (mean paired difference − 2.61; *p* = 0.0010). Subgroup analyses suggested that CRSsNP cases tended to show insufficient improvement, while younger age and higher IgE levels were associated with better response trends.

**Conclusions:**

Omalizumab significantly improved symptoms and QOL in patients with JCP. However, its high cost and the risk of nonresponse necessitate careful patient selection. Age and IgE levels may help guide treatment decisions, highlighting the importance of individualized strategies for severe JCP.

**Supplementary Information:**

The online version contains supplementary material available at 10.1186/s13223-025-00995-y.

## Background

Allergic rhinitis (AR) is characterized by a type I hypersensitivity reaction in the nasal mucosa, and sneezing, watery rhinorrhea, and nasal congestion are its three major symptoms. Japanese cedar pollinosis (JCP), caused by *Cryptomeria japonica* pollen, is a seasonal subtype of AR that is particularly prevalent in Japan. The prevalence of JCP in Japan is approximately 40%, making it a widespread health issue [1, 2]. JCP can lead to olfactory dysfunction, sleep disturbances, and decreased concentration, significantly affecting quality of life (QOL). In Tokyo, the prevalence of JCP was estimated at 45.6% in 2017, resulting in significant effects on daily life, work, and school performance. Many patients also experience sleep disturbances and emotional distress attributable to severe symptoms [3–5]. The treatment options for JCP are broadly classified into allergen avoidance, pharmacotherapy, surgical interventions, and immunotherapy.

Omalizumab (Xolair^®^), a humanized anti-immunoglobulin E (IgE) monoclonal antibody developed by Genentech in 1991, is based on a mouse monoclonal antibody targeting the Cε3 region of human IgE, with 95% of its structure being replaced by human IgG1κ [6, 7]. Omalizumab binds to free serum IgE, forming IgE–anti-IgE complexes that reduce free IgE levels, thus preventing mast cell degranulation and suppressing allergic reactions. The drug also inhibits IgE binding to dendritic cells and B cells, thereby reducing antigen presentation and IgE production [7–9]. In Japan, omalizumab has been approved for bronchial asthma since 2009 and for chronic spontaneous urticaria since 2017. In December 2019, the drug was approved for seasonal allergic rhinitis (SAR), allowing its use specifically for severe JCP [10]. However, severe JCP is uncommon outside Japan, and omalizumab has not been approved for AR in other countries, resulting in limited global clinical evidence regarding its efficacy for this indication [11]. Okubo et al. [10] described the efficacy of omalizumab in patients with severe pollinosis that was inadequately controlled by standard therapies. Their findings included significant improvements in nasal and ocular symptoms and work productivity in patients treated with omalizumab.

However, questions remain regarding the consistency and duration of omalizumab’s therapeutic effects, with some studies suggesting variability over time. In addition, nasal eosinophil testing has been explored as a potential predictor of omalizumab efficacy. Although previous studies reported high specificity and moderate sensitivity for nasal eosinophil counts in the diagnosis of AR [12, 13]. Our prior findings revealed no significant correlation between eosinophil counts and treatment response, consistent with observations that antihistamine use and the timing of sampling can affect eosinophil detection [14, 15]. Moreover, previous studies suggested that omalizumab only has a transient effect on QOL. For example, Kopp et al. reported identified a transient effect of omalizumab in pollen-allergic patients undergoing specific immunotherapy, indicating that treatment benefits might not be sustained over time [16]. Given these uncertainties, further investigation into the practical application of omalizumab for JCP is warranted.

Against this background, we aimed to evaluate the clinical efficacy of omalizumab in patients with JCP in real-world practice, focusing on clinical outcomes, QOL, and work productivity, and to explore patient characteristics associated with insufficient or non-sustained efficacy.

## Methods

### Patients and study design

This single-center, retrospective, observational analysis was conducted at the Takeda ENT Clinic (Fujimi City, Japan). Data for consecutive patients who received omalizumab for JCP during the active pollen seasons from February to April in each year of the study (2021–2024) were retrospectively reviewed.

The exclusion criteria were as follows: history of anaphylaxis or severe allergic reactions to biologic agents; presence of systemic autoimmune, infectious, or malignant diseases; poor medication adherence as judged by the treating physician; incomplete clinical records preventing retrospective evaluation; discontinuation of omalizumab within 4 weeks of initial administration; and concurrent enrollment in another interventional clinical trial.

The diagnosis of JCP was confirmed according to clinical symptoms consistent with the pollen season and positive specific IgE testing (ImmunoCAP, Thermo Fisher Scientific, Waltham, MA, USA) against *C. japonica* at class 3 or higher.

Baseline clinical characteristics, including age, sex, body weight, total serum IgE levels, specific IgE profiles (cedar, cypress, house dust mites), and comorbidities such as asthma and chronic rhinosinusitis (CRS), were documented.

The study was approved by the Ethics Committee of Nihon University School of Medicine, approval No. 2023-11). Informed consent was obtained from all participants at the time of their initial consultation to permit the use of their anonymized clinical data for research. All eligible patients received omalizumab without randomization or control group division.

## Dosage and administration of Omalizumab and efficacy assessment

Omalizumab was administered subcutaneously every 2 or 4 weeks in accordance with the approved dosing tables in Japan, which determine the dose based on each patient’s baseline total serum IgE level (30–1500 IU/mL) and body weight (20–150 kg) [10]. Patients with higher baseline IgE levels and/or greater body weight received higher doses, and the dosing interval (2 week vs. 4 week) was determined according to the same tables. The regimens used in our cohort included 150 mg, 300 mg, 450 mg, and 600 mg every 4 weeks, as well as 375 mg, 450 mg, 525 mg, and 600 mg every 2 weeks, consistent with these guidelines (Table 1).

**Table 1:** Baseline characteristics of the 42 patients treated with Omalizumab

Overall	*N* = 42
Sex (male/female)	30/12
Age, mean ± SD (range)	41.5 ± 17.2 (12–80)
Body weight (kg), mean ± SD	65.0 ± 13.5
Baseline serum total IgE levels (IU/mL) median [IQR]	95 [56–300]
Nasal eosinophil test results	1
Negative	26
1+	10
2+	2
3+	1
Specific IgE levels against cedar pollens (CAP-RAST)	
Class 2 (0.70 − 3.49 UA/mL)	0
Class 3 (3.50 − 17.49 UA/mL)	22
Class 4 (17.50 − 49.99 UA/mL)	10
Class 5 (50.00 − 99.99 UA/mL)	7
Class 6 (≥ 100 UA/mL)	3
Cypress allergy	36
Dust mite allergy	19
Comorbidities	Asthma: 2, CRSwNP: 1, CRSsNP: 2
150 mg every 4 weeks	17
300 mg every 4 weeks	11
450 mg every 4 weeks	5
600 mg every 4 weeks	2
375 mg every 2 weeks	1
450 mg every 2 weeks	1
525 mg every 2 weeks	4
600 mg every 2 weeks	1

Demographic data include sex, age, and body weight. All values are expressed as mean ± standard deviation unless otherwise indicated. Percentages are shown in parentheses. IgE, immunoglobulin E; CAP-RAST, radioallergosorbenttest; SD, standard deviation; CRS, chronic rhinosinusitis; CRSwNP, CRS with nasal polyps; CRSsNP, CRS without nasal polyps

The primary efficacy endpoint was the change in symptom severity scores from baseline to 4 weeks after omalizumab initiation, evaluated using the Japanese Rhinitis and Conjunctivitis Quality of Life Questionnaire [17]. Treatment response was also assessed by patient-reported questionnaire at 4 weeks. For all patients (*N* = 42; 2021–2024), responses were classified into three-categories: “marked improvement,” “improvement with residual symptoms,” or “no improvement”. In the 2023–2024 subset (*n* = 23), quantitative total symptom score (TSS) data were additionally available, enabling effect-size estimation, subgroup analyses, and logistic regression modeling. In addition to symptom assessment, the following parameters were evaluated: blood test results, including total IgE and eosinophil counts; nasal eosinophil test results, graded as negative, + 1, +2, or + 3; medication score, reflecting the reduction in concomitant medication usage; and impact on daily life (concentration at work or during study, fatigue or frequent tiredness, difficulty falling asleep, frequent nighttime awakening, other patient-reported symptoms).

Symptom scores were recorded on a 5-point scale (0–4) for both sneezing and nasal blowing frequency (0 times, 0 points; 1–5 times, 1 point; 6–10 times, 2 points; 11–20 times, 3 points; and ≥ 21 times, 4 points) and nasal congestion (no congestion, 0 points; mild congestion without mouth breathing, 1 point; moderate congestion requiring occasional mouth breathing, 2 points; severe congestion requiring frequent mouth breathing; 3 points; and complete nasal obstruction, 4 points).

### Statistical analysis

Statistical analyses were performed using RStudio (version 4.1.1; R Foundation for Statistical Computing, Vienna, Austria). Normally distributed variables are presented as mean ± SD, and non-normally distributed variables as median (IQR). Given the relatively small sample size and the nonparametric distribution of most variables, the Wilcoxon signed-rank test was primarily used to compare pre- and post-treatment values.

Despite its known limitations associated with small sample datasets, analysis of variance was additionally applied for descriptive purposes to detect any potential trends or differences across multiple symptom subgroups (e.g., stratified by age groups, total IgE levels). To explore baseline predictors of nonresponse, we performed univariable logistic regression analysis with Firth’s penalized likelihood, including variables such as age, sex, body weight, total IgE, cedar/cypress-specific IgE, nasal eosinophil status, dosing regimen, and comorbidities (asthma, CRSwNP, CRSsNP).

The threshold for statistical significance was set at *p* < 0.05 and F < 0.05.

## Results

### Patient characteristics

The characteristics of the study population are summarized in Table 1. In total, 42 patients who received omalizumab between January 2021 and May 2024 and met the inclusion and exclusion criteria were included in this analysis. The cohort included 30 males (71.4%) and 12 females (28.6%) with a mean age of 41.5 ± 17.2 years (range, 12–80 years) and a mean body weight of 65.0 ± 13.5 kg.

The median baseline serum total IgE level of the study population was 95 IU/mL (IQR, 56–300).The nasal eosinophil test results were negative in 26 patients, + 1 in 10 patients, + 2 in 2 patients, + 3 in 1 patient, and unknown in 3 patients. The mean specific IgE level against cedar pollen was 31.3 ± 30.3 UA/mL.

Asthma was observed in 2 patients (4.8%). Chronic rhinosinusitis (CRS) was present in 3 patients (7.1%), including 1 patient (2.4%) with CRS with nasal polyps (CRSwNP) and 2 patients (4.8%) with CRS without nasal polyps (CRSsNP).

The distribution of omalizumab dosing regimens was as follows: 150 mg every 4 weeks, 17 patients; 300 mg every 4 weeks, 11 patients; 450 mg every 4 weeks, 5 patients; 600 mg every 4 weeks, 2 patients; 375 mg every 2 weeks, 1 patient; 450 mg every 2 weeks, 1 patient; 525 mg every 2 weeks, 4 patients; and 600 mg every 2 weeks, 1 patient.

### Treatment response

Among the entire cohort (*N* = 42; 2021–2024), symptom improvement was observed in 38 patients (90%), including marked improvement in 23 patients (55%) and improvement with residual symptoms in 15 patients (36%), whereas four patients (10%) experienced no improvement.

**Fig. 1:**
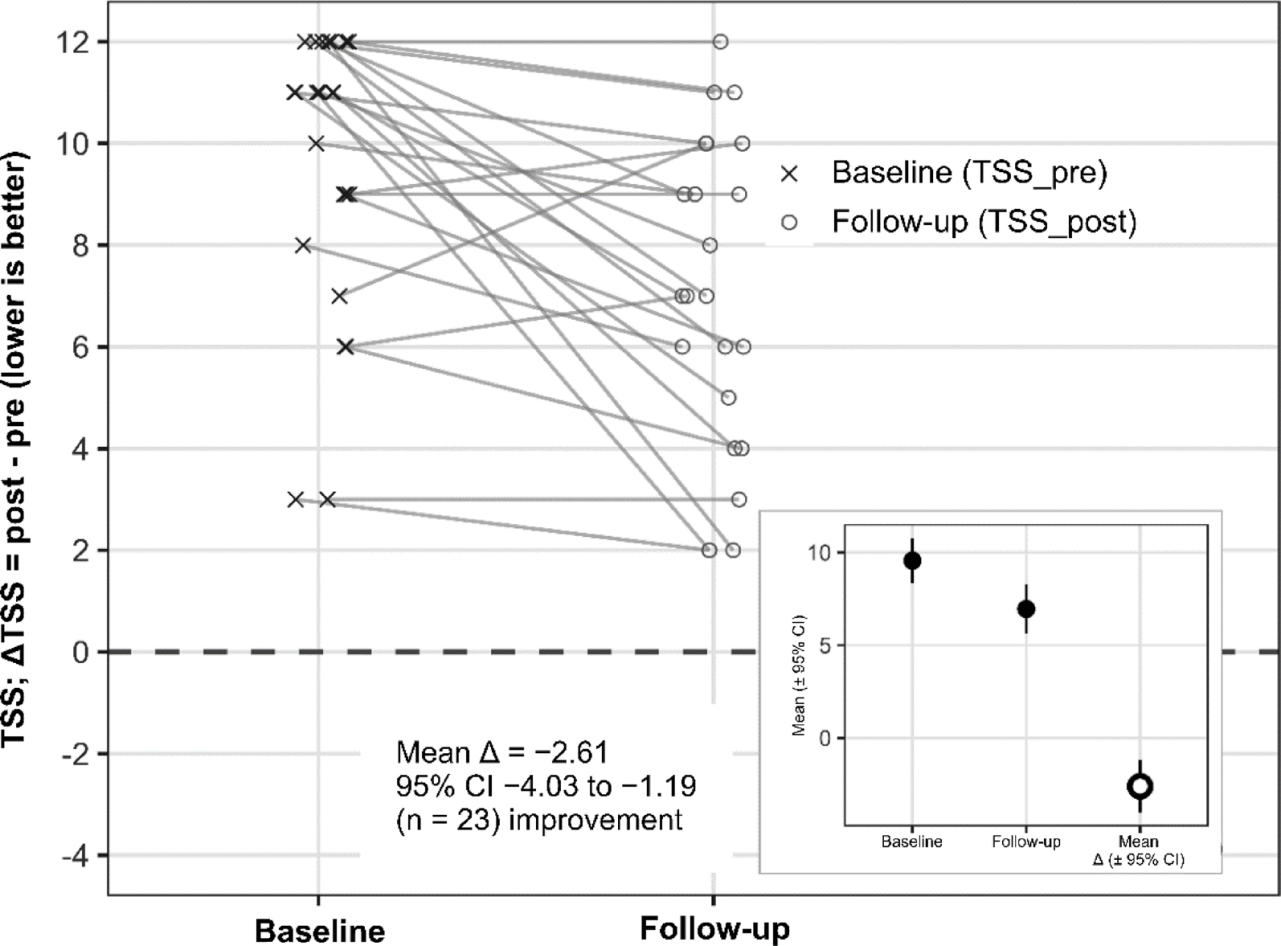
Changes in Total Symptom Score (TSS) Before and AfterOmalizumab Treatment in the 2023–2024 Cohort (*n* = 23)

Of note, treatment response was uniformly assessed using a 3-category patient-reported questionnaire during 2021–2024. In a subset of patients treated in 2023–2024 (*n* = 23), TSS data were additionally available, enabling effect-size estimation and subgroup analyses (Table 3). The changes in sneezing, nasal congestion, and rhinorrhea between 2023 and 2024 are illustrated in Fig. 1. Significant symptom improvement was noted following omalizumab administration (all *p* < 0.05). In the 2023–2024 subset (*n* = 23, no missing data), TSS decreased significantly, with a mean paired difference of − 2.61 (95% CI, − 4.03 to − 1.19; *p* = 0.0010, paired t-test). A nonparametric analysis confirmed a Hodges–Lehmann pseudo-median change of − 2.50 (95% CI, − 4.00 to − 1.00; *p* = 0.0013). Component-wise, sneezing improved by a pseudo-median of − 1.00 (*p* = 0.0008; Holm-adjusted *p* = 0.0025; rank-biserial *r* = − 0.91), nasal obstruction by − 1.00 (*p* = 0.0090; Holm-adjusted *p* = 0.0179; *r* = − 0.71), and rhinorrhea by − 0.50 (*p* = 0.0099; Holm-adjusted *p* = 0.0099; *r* = − 0.91).

**Table 2:** Baseline characteristics of responders and nonresponders to Omalizumab

Overall (*n* = 42)	Symptom improvement (*n* = 38)	No improvement (*n* = 4)
Rate (%)	90	10
Age (years)	39.9	55.5
IgE (IU/mL)	251	211
Omalizumab Dose (mg)	310	300
Body weight (kg)	65	64
Specific IgE levels against cedar pollens (CAP-RAST) (UA/mL)	31.8	26.9

Responders (*n* = 38) were younger, had higher total and specific IgE levels, and were administered slightly higher omalizumab doses than nonresponders (*n* = 4). Symptom improvement was observed in 90% of patients overall. IgE, immunoglobulin E ; SD, standard deviation; UA, allergen specif icIgE unit (used in CAP RAST)

A comparison of total serum IgE levels revealed that responders group had higher IgE levels than nonresponders (251.1 IU/mL vs. 210.5 IU/mL; normal, ≤ 170 IU/mL). The mean age was 39.9 years among responders, versus 55.5 years among nonresponders. The specific IgE levels against cedar pollen were 31.8 UA/mL in responders and 26.9 UA/mL in nonresponders (Table 2).

**Table 3:** Subgroup analyses of treatment response based on comorbidities (*n* = 23; 2023–2024, TSS-based evaluation)

Subgroup	n_with	Denominator	Prevalence %	With_resp *n*/*N* (%)	Without_resp *n*/*N* (%)	OR (95% CI)	Fisher *p*	Holm *p*	ΔTSS MW *p*
CRSwNP	1	23	4.3	1/1 (100.0)	9/22 (40.9)	4.26 (0.16–116.34)	0.435	1	1
CRSsNP	1	23	4.3	0/1 (0.0)	10/22 (45.5)	0.40 (0.01–10.80)	1	1	0.172
CRS overall	2	23	8.7	1/2 (50.0)	9/21 (42.9)	1.33 (0.07–24.32)	1	1	0.35
Asthma	2	23	8.7	1/2 (50.0)	9/21 (42.9)	1.33 (0.07–24.32)	1	1	0.7

This table summarizes the prevalence of comorbid conditions and treatment responder rates, stratified by CRS with and without nasal polyps, and asthma. Responders were defined as patients achieving a ≥ 30% reduction in TSS. No between-group differences reached statistical significance. CRS, chronic rhinosinusitis; CRSwNP, CRS with nasal polyps; CRSsNP, CRS without nasal polyps; OR, odds ratio; CI, confidence interval; TSS, total symptom score

**Fig. 2:**
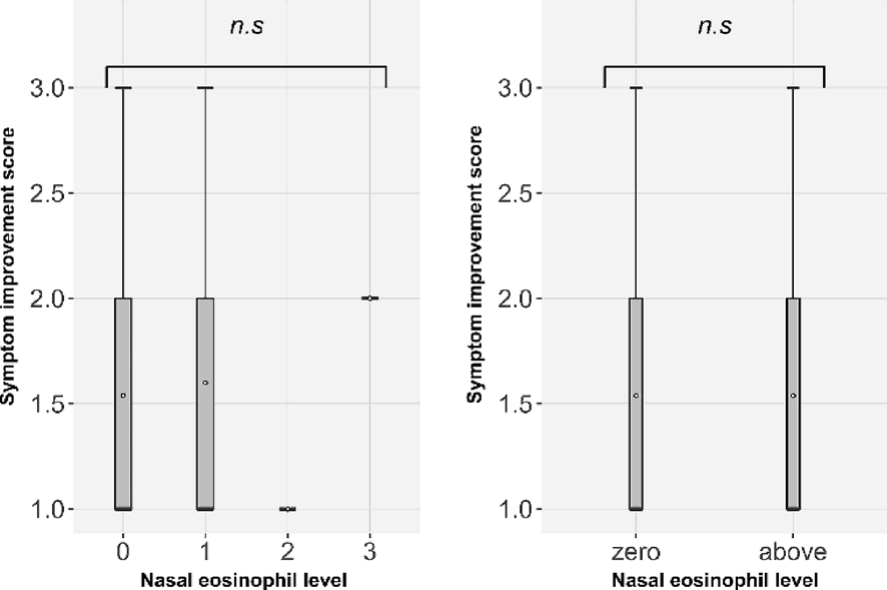
Correlations between nasal eosinophil test results and the degree of symptom improvement following omalizumab treatment

There was no significant correlation between nasal eosinophil counts and symptom improvement following omalizumab treatment (F = 0.96, *p* = 0.986; Fig. 2). We further examined the prevalence of comorbidities and their potential impact on treatment response, with responders defined as those achieving ≥ 30% reduction in TSS (Table 3). Subgroup analyses showed the following: CRSwNP, 1/1 (100.0%) vs. 9/22 (40.9%) (OR 4.26 [0.16–116.34], Fisher *p* = 0.435, Holm-adjusted *p* = 1.000); CRSsNP, 0/1 (0.0%) vs. 10/22 (45.5%) (OR 0.40 [0.01–10.80], *p* = 1.000, Holm-adjusted *p* = 1.000); CRS overall, 1/2 (50.0%) vs. 9/21 (42.9%) (OR 1.33 [0.07–24.32], *p* = 1.000, Holm-adjusted *p* = 1.000); and asthma, 1/2 (50.0%) vs. 9/21 (42.9%) (OR 1.33 [0.07–24.32], *p* = 1.000, Holm-adjusted *p* = 1.000). Between-group differences in ΔTSS (Mann–Whitney test) were not significant (CRSwNP *p* = 1.000, CRSsNP *p* = 0.172, CRS overall *p* = 0.350, asthma *p* = 0.700). None of these subgroup analyses demonstrated statistically significant differences, although patients with CRSsNP tended to show insufficient improvement. To further explore baseline predictors of nonresponse, univariable logistic regression analysis with Firth’s penalized likelihood was performed in the overall cohort (*n* = 42) using the categorical patient-reported classification of treatment response (Table 4). Older age tended to increase the odds of nonresponse, whereas the association with total IgE was unstable and did not reach statistical significance. Nasal eosinophil positivity also showed a nonsignificant trend toward nonresponse. No clear associations were found for sex, body weight, cedar/cypress-specific IgE, dosing regimen, or comorbidities.

**Table 4:** Univariable logistic regression analysis of factors associated with nonresponse to Omalizumab (*n* = 42, based on categorical treatment response classification)

Variable	OR	95% CI	*p*-value	*n*
Age (per 10 years)	1.69	0.90–3.18	0.102	42
Male vs. Female	1.56	0.15–16.53	0.712	42
Body weight (per 5 kg)	0.85	0.66–1.09	0.205	42
Total IgE (log10; per 10-fold)	1.48	0.16–13.67	0.728	42
Cedar-specific IgE (per 10 UA/mL)	0.99	0.70–1.40	0.96	42
Cedar class	0.67	0.19–2.41	0.54	42
Cypress class	1	1.00–1.01	0.755	42
Omalizumab dose ≥ 300 mg versus < 300 mg	0.37	0.04–3.89	0.408	42
Dosing interval 2w versus 4w	2.66E-18	0.00–inf	1	42
Nasal eosinophils test positive versus negative	2.45	0.31–19.68	0.398	42

OR, odds ratio; CI, confidence interval. Univariable logistic regression analyses were conducted using Firth’s penalized likelihood correction, due to the small number of nonresponders (*n* = 4) in the overall cohort (*n* = 42). An OR > 1 indicates increased odds of nonresponse, while an OR < 1 indicates decreased odds. Although older age showed a nonsignificant trend toward higher odds of nonresponse, and total IgE had an OR > 1, both estimates were unstable, with wide confidence intervals and no statistical significance. Therefore, no definitive associations can be concluded for IgE or any of the other variables analyzed, including body weight, sex, cedar/cypress-specific IgE, nasal eosinophil status, omalizumab dose, or dosing interval

**Fig. 3:**
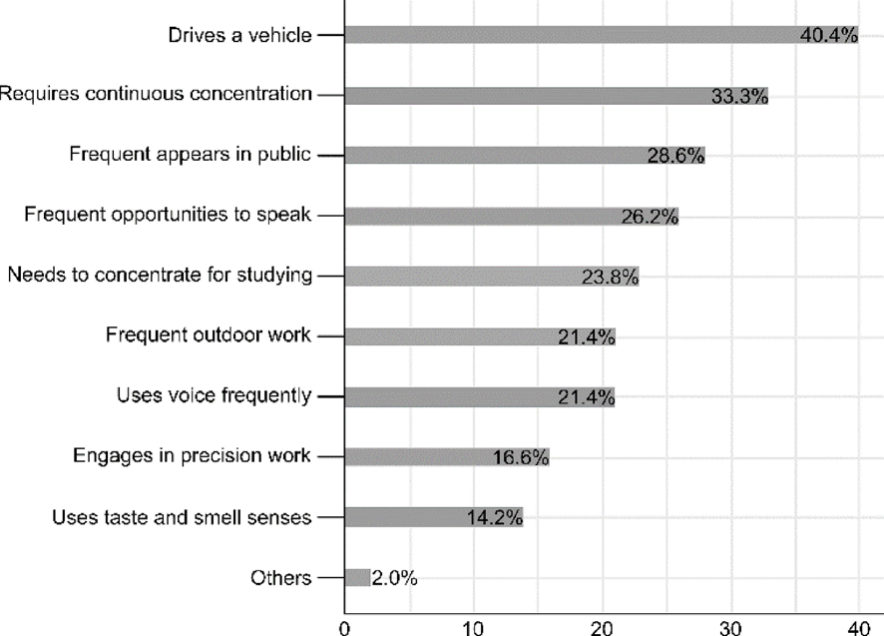
Patient-reported daily life burdens before omalizumab treatment

**Fig. 4:**
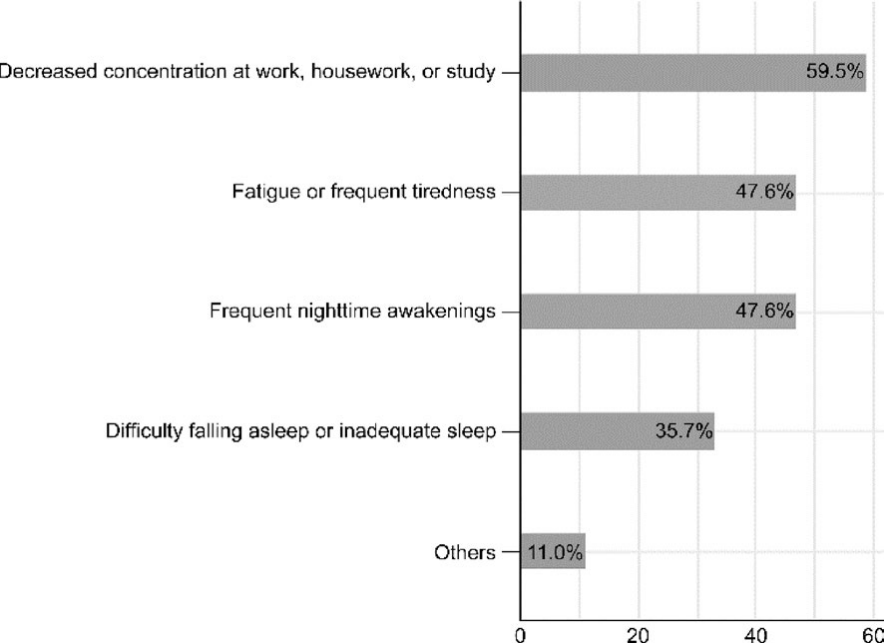
Occupational and activity requirements before omalizumab treatment

In addition to symptom improvement, we examined the impact of JCP on patients’ daily life and occupational demands prior to treatment. These findings are summarized in Figs. 3 and 4.

## Patients’ pretreatment concerns and lifestyles

The patients’ concerns regarding their daily lives before treatment included decreased concentration at work, housework, or study (25 patients [59.5%]), fatigue or frequent tiredness (20 patients [47.6%]), frequent nighttime awakening (20 patients [47.6%]), and difficulty falling asleep or inadequate sleep (15 patients [35.7%]; Fig. 3).

Regarding pretreatment lifestyle factors, the most common occupational and activity-related requirements were “drives a vehicle” (17 patients [40.4%]), “requires continuous concentration” (14 patients [33.3%]), “frequent appearance in public” (12 patients [28.6%]), and “frequent opportunities to speak” (11 patients [26.2%]; Fig. 4).

## Discussion

### Efficacy of Omalizumab in our study

In our study, omalizumab achieved a high rate of symptom improvement in patients with JCP, with most patients experiencing marked improvement. This finding is consistent with those of Okubo et al. [10, 11] in patients with JCP and Goto et al. [18] in patients with severe cedar pollen-induced SAR.

Interestingly, our data indicated that younger patients exhibited a better response to treatment than older individuals. One possible explanation for this finding is that age-related changes in nasal epithelial function can diminish the effectiveness of IgE-targeted therapy [19–21].

Additionally, higher total serum IgE levels were associated with better treatment responses. However, the limitations of conventional assays such as ImmunoCAP, which cannot differentiate free IgE from omalizumab-bound IgE, must be considered [22, 23]. Future evaluations incorporating measurements of serum free IgE might better predict treatment responsiveness.

### Nasal eosinophil testing and predictive value

We assessed whether nasal eosinophil positivity could predict the clinical response to omalizumab. However, no significant correlation was observed between nasal eosinophil counts and symptom improvement. This aligns with previous findings by Hirano et al. [24], who also reported that nasal eosinophil counts were not strongly predictive of therapeutic outcomes in patients who received omalizumab for severe pollinosis. Factors such as the timing of sampling and antihistamine use could have affected eosinophil detection, highlighting the need for more standardized collection methods in future research.

In contrast, younger age and higher total serum IgE levels were associated with greater symptom improvement, suggesting that these factors may serve as useful predictors of omalizumab efficacy. Patients with comorbid CRSsNP showed insufficient improvement, consistent with evidence that disease phenotypes can influence therapeutic outcomes [28]. Although the relatively small sample size precluded multivariate modeling, future studies with larger cohorts should incorporate variables such as IgE, age, nasal eosinophils, and comorbidities into predictive models to better identify patients most likely to benefit from omalizumab. Univariable logistic regression analysis suggested that older age tended to increase the odds of nonresponse, whereas the association with total IgE was unstable across analytic scales. For total IgE, the odds ratio exceeded 1; however, the estimate was imprecise, with a very wide confidence interval, and did not reach statistical significance. Nasal eosinophil positivity also showed a nonsignificant trend toward nonresponse, while body weight, sex, dosing regimen, and comorbidities demonstrated no clear associations.

### Impact of JCP on daily life and QOL improvement

Prior to treatment, our patients reported substantial impairments in daily functioning, including decreased concentration, fatigue, and sleep disturbances.

These results were in line with previous observations, in which more than 80% of patients with moderate-to-severe AR reported significant daily life and emotional impacts [25]. Following omalizumab administration, many patients—especially students preparing experienced marked improvements in symptom control during critical periods. Our findings are comparable to those of Kopp et al. [16], who demonstrated that omalizumab transiently improved QOL life in pollen-allergic patients undergoing specific immunotherapy. In addition, a recent comparative study by Tang et al. [26] reported that omalizumab therapy provided comparable or superior symptom control and QOL improvement as conventional pharmacotherapy and specific immunotherapy in patients with hay fever.

These results underscore the potential utility of omalizumab in improving both nasal symptoms ad overall QOL, particularly during high-demand periods.

### Clinical safety of Omalizumab

No serious adverse events related to omalizumab were observed in our cohort, with the most common complaint being localized injection site pain (data not shown). This safety profile is consistent with those of prior studies, including those by Okubo et al. [3] and Kopp et al. [16], who found that omalizumab was generally well tolerated in patients with severe AR or pollen allergies.

### Impact of asthma comorbidity

In our cohort, 2 patients had asthma as a comorbidity. Both cases were well controlled with oral medications (a leukotriene receptor antagonist and an antihistamine) did not require inhaled corticosteroids. According to the Global Initiative for Asthma (GINA) 2024 classification, these cases would be categorized as mild asthma [27]. The presence of mild, well-controlled asthma did not appear to influence the clinical response to omalizumab in this study.

It is worth noting that omalizumab was originally developed and approved for the treatment of severe allergic asthma. Therefore, asthma comorbidity could theoretically affect treatment outcomes. However, since both asthma cases in our cohort were mild and managed without inhaled therapy, the observed symptom improvement is unlikely to have been confounded by omalizumab’s established efficacy in severe asthma.

### Nonresponders and future considerations

Nonetheless, omalizumab treatment was ineffective in approximately 10% of our patients. Nonresponse was generally observed among older individuals, those with multiple allergen sensitizations, and those with comorbid CRS without nasal polyps. This is consistent with the findings of phase 3 trials by Gevaert et al. [28], who demonstrated that omalizumab is more effective in patients with nasal polyposis, whereas its efficacy might be limited in patients with nonpolyp CRS phenotypes.

These findings underscore the importance of careful patient selection to optimize cost-effectiveness, particularly considering the high cost of biologic therapies.

Moreover, emerging evidence suggests that omalizumab might have broader clinical applications beyond SAR. A recent study by Ren et al. [29] demonstrated the efficacy of omalizumab against various IgE-mediated allergic diseases, highlighting its potential versatility in managing complex allergic conditions. In addition, previous studies, such as that by Kuehr et al. [30], described the benefits of combining omalizumab with allergen-specific immunotherapy in polysensitized patients.

These findings suggest that omalizumab could be incorporated into combination therapeutic strategies, particularly for patients with multiple sensitization or refractory disease, thereby expanding its potential clinical utility in the future.

With future advancements in biotechnology, the cost of antibody production might decrease, and the safety profile of biologic therapies might be further improved. If these advancements are realized, then the treatment options for pollinosis in Japan could expand significantly. Furthermore, in addition to JCP, omalizumab might emerge as a viable general treatment option for AR in the future.

### Study limitations

This study has several limitations. First, the sample size was relatively small, and the data were limited to a single-center cohort, which may restrict the generalizability of the findings. This small sample size may also have reduced the statistical power of our analyses and should be taken into account when interpreting the results. Second, as a retrospective study, the data may have been affected by incomplete records or selection bias. Third, symptom severity and treatment efficacy were primarily assessed based on patient self-reports, which may introduce subjectivity. Treatment response was classified into three patient-reported categories (“marked improvement,” “improvement with residual symptoms,” or “no improvement”) without predefined numerical thresholds, potentially limiting reproducibility. Additionally, peripheral blood eosinophil counts were not included routinely collected before omalizumab administration, preventing assessment of pre- and post-treatment changes and limiting the biological interpretation of treatment response. Furthermore, evaluation of nasal eosinophils may have been influenced by factors such as the timing and method of sample collection as well as concomitant medication use. Future prospective, multicenter studies using standardized assessment protocols are needed to validate and expand upon these findings. Moreover, response was assessed using a three-category patient-reported scale across all patients from 2021 to 2024. In a subset of patients treated in 2023–2024 (*n* = 23), TSS data were additionally available, enabling effect-size estimation and subgroup analyses; however, this heterogeneity in outcome availability limited uniform calculation of effect sizes across the entire cohort.

Additionally, predictors of nonresponse were explored using univariable logistic regression in the overall cohort (*n* = 42); however, given the very limited number of nonresponders (*n* = 4), the resulting estimates were unstable and should be regarded as exploratory.

Finally, this study did not include post-treatment assessments of patients’ daily concerns, lifestyles, occupational demands, or activity requirements. Evaluations after omalizumab therapy were limited to nasal symptoms and overall treatment efficacy, and the lack of post-treatment lifestyle data may restrict understanding of the broader real-world impact of omalizumab. Future studies incorporating these aspects will be important to provide a more comprehensive assessment of patient outcomes.

### Summary and implications

Thus, our findings indicate that omalizumab significantly improved symptoms and QOL in patients with JCP, particularly among younger individuals and those with higher total IgE levels. However, approximately 10% of patients did not respond to treatment, with nonresponse more frequently observed in older adults and those with multiple allergen sensitizations or comorbid CRS. Given the high cost of omalizumab, careful patient selection based on clinical characteristics is essential to ensure its optimal and cost-effective application in clinical practice.

This study underscores the importance of individualized treatment strategies in the management of severe JCP. Further research is warranted to better understand the long-term efficacy of omalizumab, its role in patients with comorbidities, and the potential benefits of combining omalizumab with other therapies, such as sublingual immunotherapy. One additional limitation of this study is that treatment response was defined using patient-reported categories rather than quantitative thresholds of symptom reduction, which may introduce subjectivity in efficacy assessment.

## Conclusion

Omalizumab significantly improved symptoms and QOL in patients with severe JCP. Individualized treatment strategies are essential, and further research is needed to clarify its long-term efficacy and role in patients with comorbidities.

Lines represent individual patients’ TSS at baseline and 4 weeks post-treatment. Crosses indicate individual scores; the diamond represents the mean change (ΔTSS) with 95% confidence intervals (CI). The mean paired difference was − 2.61 (95% CI, − 4.03 to − 1.19; *p* = 0.0010), indicating significant improvement. Negative values reflect symptom reduction.

The left panel compares the eosinophil-negative, + 1, +2, and + 3 groups using analysis of variance (F = 0.96).

The right panel compares eosinophil-negative and eosinophil-positive patients (+ 1–+3; Wilcoxon’s signed-rank test, *p* = 0.986).

This figure illustrates the self-reported burdens of patients with severe seasonal allergic rhinitis before omalizumab initiation. The most commonly reported issues included reduced concentration at work, study, or household tasks, along with fatigue and sleep disturbances. Multiple responses were allowed.

This figure shows the specific occupational and lifestyle-related demands of patients before treatment. Many reported the need to drive, maintain continuous concentration, appear in public, or engage in frequent verbal communication. Understanding these factors helps assess the broader impact of allergic rhinitis on daily functioning.

## Supplementary Information

Below is the link to the electronic supplementary material.

Supplementary Material 1

## Data Availability

No datasets were generated or analysed during the current study.

## Abbreviations

JCP: Japanese cedar pollinosis
QOL: quality of life
IgE: immunoglobulin E
AR: allergic rhinitis
SAR: seasonal allergic rhinitis
CRS: chronic rhinosinusitis
CRSwNP: chronic rhinosinusitis with nasal polyps
CRSsNP: chronic rhinosinusitis without nasal polyps
