# Supplementary material for: Efficacy of Omalizumab against Japanese Cedar pollinosis in clinical practice
Source: Allergy Asthma Clin Immunol. 2025 Nov 28;21:50. doi: 10.1186/s13223-025-00995-y (PMC12664126; doi:10.1186/s13223-025-00995-y)

**Supplementary Figure 1. Symptom-specific changes following omalizumab therapy.**

Changes in sneezing, nasal congestion, and rhinorrhea scores before and after 4 weeks of omalizumab treatment are shown. Significant improvements were observed in sneezing and nasal congestion, with a trend toward improvement in rhinorrhea.


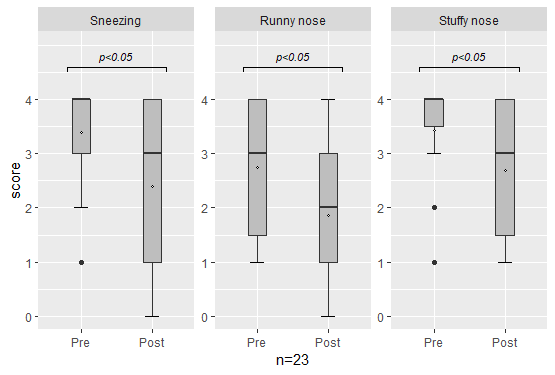

Supplement: Supplementary file 1 — Supplementary Material 1 [file 13223_2025_995_MOESM1_ESM.docx]
